# Contralateral Axillary Lymph Node Metastasis of Breast Cancer: Retrospective Analysis and Literature Review

**DOI:** 10.3389/fonc.2022.869397

**Published:** 2022-04-14

**Authors:** Liang Zhang, Xin zhao Wang, Chao Li, Qian Yu, Zhaoyun Liu, Zhi yong Yu

**Affiliations:** ^1^ Department of Breast Surgery, Shandong Cancer Hospital and Institute, Shandong First Medical University and Shandong Academy of Medical Sciences, Jinan, China; ^2^ Interventional Radiology, University of Chicago, Chicago, IL, United States

**Keywords:** contralateral axillary lymph node metastasis (CAM), breast cancer staging, local recurrence, treatment strategy, breast carcinoma (BC)

## Abstract

**Background:**

Contralateral axillary lymph node metastasis (CAM) is classified as distant metastasis in guidelines, but the prognosis is better than that of stage IV patients. It is controversial to classify CAM as a distant metastasis or a regional metastasis, and the optimal treatment strategy for CAM is unknown.

**Patients and Methods:**

Breast cancer patients who were confirmed by pathology and treated at Shandong Cancer Hospital between January 2012 and July 2021 were included in our study. We retrospectively reviewed the medical records of the patients for their clinical features, pathological diagnosis, treatment strategy, and follow-up data. Survival analysis was calculated by Kaplan–Meier analysis, and patient matching was performed by case–control matching.

**Results:**

A total of 60 patients were included, and there were 49 metachronous CAM cases and 11 synchronous CAM cases. The prognosis of isolated CAM patients was better than that of patients with other distant metastases in terms of CAM-OS and PFS with significant differences (median CAM-OS 71.0 vs. 30.0 months, P=0.022; median PFS 42.0 vs. 11.0 months, P=0.009) and OS without significant differences (median OS 126.0 vs. 79.0 months, P=0.111). The five-year survival rate of isolated CAM patients was 67.4%, and the five-year disease-free survival (DFS) rate was 52.9%. The prognosis of CAM patients was similar to that of N3M0 patients in terms of OS (mean OS 82.4 vs. 65.6 months, P=0.537) and DFS (mean PFS 54.5 vs. 52.6 months, P=0.888). Axillary lymph node dissection (ALND) or low-middle level ALND significantly improved the OS (mean OS 237.4 vs. 111.0 months, P=0.011), CAM-OS (mean CAM-OS 105.2 vs. 46.6 months, P = 0.002), and PFS (mean PFS 92.3 vs. 26.9 months, P = 0.001) of isolated CAM patients. Axillary radiotherapy improved PFS, CAM-OS, and OS but without significant differences (mean PFS 80.0 vs. 46.6 months, P = 0.345; mean CAM-OS 86.8 vs. 72.1 months, P = 0.338; mean OS 147.6 vs. 133.0 months, P = 0.426).

**Conclusion:**

CAM should be diagnosed as local recurrence and treated with aggressive and curative rather than palliative strategies. Contralateral axillary surgery and radiotherapy are recommended for isolated CAM patients.

## Introduction

The presence of contralateral axillary lymph node metastasis (CAM) without other organ involvement in breast cancer is rare with a reported incidence ranging between 0.81 and 6% of the total population (1–5).

The regional lymph nodes of the breast include the ipsilateral axillary, subclavian, supraclavicular, and internal mammary lymph nodes, but contralateral axillary lymph nodes are not included. CAM larger than 0.2 mm is classified as M1 (stage IV) rather than stage III according to the TNM classification in the seventh edition of the American Joint Commission on Cancer (AJCC) (6). However, the prognosis of CAM patients is better than that of stage IV patients (7).

The optimal treatment strategies are controversial, especially when CAM is the primary event of recurrence after primary tumor treatment. There is no standard treatment guideline for CAM, and patients need individualized treatment. At present, the impact of different treatment strategies on the prognosis of CAM is not clear. To date, the relevant literature consists of small-scale studies or case reports, and the details and integrity of the literature data vary greatly (8).

The mechanism of isolated CAM is different from that of CAM with ipsilateral mammary recurrence, and the occurrence of isolated CAM occurs much earlier. Isolated CAM may be an occult contralateral nodal metastasis of the primary breast cancer remaining *in situ* during the treatment, while CAM with ipsilateral mammary recurrence should be regarded as a regional metastasis recurrent breast tumor (8).

Whether CAM is regarded as a distant metastasis or a regional metastasis to the contralateral breast is currently controversial. There is a lack of large-scale clinical studies on the treatment and prognosis of CAM due to its low morbidity. It is difficult to develop a treatment strategy when CAM is the first event after treatment failure of the primary tumor, especially without other distant organ metastasis. In the present study, we aimed to evaluate the clinicopathologic characteristics of the tumor and the prognosis of patients who suffered from CAM, and we also aimed to clarify the stage and therapeutic approaches of CAM.

## Materials and Methods

The present study was a single-center, retrospective study. Breast cancer patients who were confirmed by pathology and treated at Shandong Cancer Hospital between January 2012 and July 2021 were included in our study. Patients who were initially diagnosed as N3M0 and CAM patients were included in the study. CAM was defined as synchronous CAM if the cases were diagnosed at the same time as the primary tumor or within 1 year after the initial diagnosis of the primary tumor. If CAM was detected over 1 year after the initial diagnosis of the primary tumor, we defined the cases as metachronous CAM. The diagnostic methods of CAM included pathological diagnosis of operation/biopsy, fine needle aspiration cytology, and imaging diagnosis. In addition to the contralateral axillary lymph nodes, patients with metastasis of other sites were also included in this study. The clinical, pathological, and prognostic data of all patients were collected in this study.

Estrogen receptor (ER) and progesterone receptor (PR) testing was performed by immunohistochemistry (IHC). Cancers with 1%–100% of cells positive for ER/PR expression were considered ER-/PR-positive, and cancers with <1% staining were considered negative. HER2 testing was performed using methodology outlined in the ASCO/CAP HER2 testing guideline.

Continuous data are expressed as medians and intervals, and categorical data are expressed as counts and percentages. The therapeutic effect was evaluated by overall survival (OS), overall survival after CAM diagnosis (CAM-OS), disease-free survival (DFS), and progression-free survival (PFS). Cam-OS was defined as the time from the diagnosis of CAM to death. Survival analysis was calculated by Kaplan–Meier analysis. Case–control matching was performed by molecular type, year of diagnosis, and age of diagnosis.

## Results

### Initial Clinic-Pathological Characteristics and Metastasis

A total of 60 CAM patients were selected from 1247 advanced breast cancer patients in this study. The clinical and pathological characteristics at the time of the initial diagnosis are summarized in **Table 1**.

**Table 1:** Clinical and pathological characteristics of patients at initial diagnosis.

Variable	Number	Percent
Primary Side of Tumor	60	
Left	47	78.3%
Right	13	21.7%
Menstrual status	60	
Menopausal	18	30.0%
Premenopausal	42	70.0%
Primary Tumor Location	19	
Inner Upper Quadrant	2	10.5%
Outer Lower Quadrant	4	21.1%
Outer Upper Quadrant	9	47.4%
Central Region	3	15.8%
Inflammatory Breast Cancer	1	5.3%
Histopathological Grade	26	
I	0	0
II	14	53.8%
II-III	4	15.4%
III	8	30.8%
Stage	49	
I	0	0
IIa	3	6.1%
IIb	7	14.3%
IIIa	12	24.5%
IIIb	3	6.1%
IIIc	22	44.9%
IV	2	4.1%
T Stage	49	
0	1	2.0%
1	3	6.1%
2	23	46.9%
3	6	12.2%
4	16	32.7%
N Stage	54	
0	5	9.3%
1	12	22.2%
2	11	20.4%
3	26	48.1%
Molecular Subtype	49	
Luminal A	10	20.4%
Luminal B	9	18.4%
HER2 Enriched	15	30.6%
Triple Negative	15	30.6%

**Figure 1:**
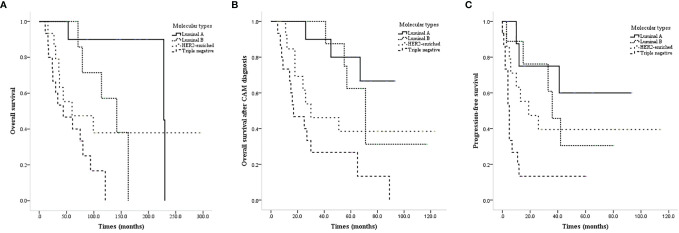
Survival curves of CAM patients based on different molecular types at the initial diagnosis. **(A)** OS of CAM patients based on different molecular types at the initial diagnosis. **(B)** CAM-OS of CAM patients based on different molecular types at the initial diagnosis. **(C)** PFS of CAM patients based on different molecular types at the initial diagnosis.

All of the patients were female. The onset age ranged from 23 to 69 years, and the median/mean onset age was 44.0/44.9 years. The primary tumor pathological type was definite in 49 patients, including 42 invasive ductal carcinomas, 1 myeloid carcinoma, 2 lipid secreting carcinomas, and 4 invasive lobular carcinomas. There was a significant difference in OS, CAM-OS, and PFS among patients with different molecular types at the initial diagnosis (P<0.001, P=0.003, and P=0.001, respectively; **Figures 1A**–**C**). 

There were 49 metachronous CAMs and 11 synchronous CAMs. The median time from the initial diagnosis to the occurrence of CAM was 30.5 months, and the mean time was 45.5 months (range 0-185 months), including 2 cases diagnosed with CAM at the initial diagnosis, 4 cases diagnosed with CAM within 6 months, and 11 cases diagnosed with CAM within 12 months.

The molecular types of the contralateral axillary lymph nodes were definite in 17 cases (**Table 2**).

**Table 2:** Molecular types of primary tumors and contralateral axillary lymph nodes.

	ER%	PR%	HER2	Ki67%	Molecular Type	CAM-ER%	CAM-PR%	CAM-HER2	CAM-Ki67%	Molecular Type
**1**	—	—	—	10	Triple Negative	—	—	—	5	Triple Negative
**2**	—	—	—	90	Triple Negative	—	—	—	90	Triple Negative	
**3**	—	—	—	50	Triple Negative	—	—	—	85	Triple Negative
**4**	95	20	1+	30	Luminal B	—	—	—	30	Triple Negative
**5**						—	—	—		Triple Negative
**6**	30	50	—	15	Luminal A	80	—	1+	30	Luminal B
**7**						90	30	—	50	Luminal B
**8**	10	10	—	50	Luminal B	70	70	—	40	Luminal B
**9**	60	80	1+	10	Luminal A	50	60	1+	65	Luminal B
**10**	90	90	—	45	Luminal B	90	—	—	40	Luminal B
**11**	80	20	—	10	Luminal A	90	—	—	35	Luminal B
**12**	50	60	—	40	Luminal B	70	60	1+	50	Luminal B
**13**						—	60	1+	75	Luminal B
**14**	—	—	3+		HER2-Enriched	—	—	3+	60	HER2-Enriched
**15**	—	—	3+	15	HER2-Enriched	—	—	3+	60	HER2-Enriched
**16**	—	—	3+	35	HER2-Enriched	—	—	3+	15	HER2-Enriched
**17**	10	—	1+	30	Luminal B	—	—	3+	30	HER2-Enriched

At the time of initial diagnosis, 34 patients had isolated CAM without other distant metastasis, and 26 patients had complicated other distant metastasis, including 17 patients with bone metastasis, 4 patients with lung metastasis, 3 patients with brain metastasis, and 11 patients with liver metastasis.

### Treatment for CAM and Prognosis

There were 2 patients who refused any treatment after CAM. Of the remaining cases, 52 patients received chemotherapy, 8 patients received anti-HER2 therapy, 6 patients received contralateral axillary radiotherapy, and 16 patients received endocrine therapy. A total of 20 patients underwent contralateral axillary lymph node dissection (ALND) or low-middle level ALND, and 3 patients underwent surgical castration. Detailed information on the pathological results of lymph nodes at different levels was queried in 12 patients (**Table 3**). Contralateral mastectomy was performed in 5 patients, and no tumor was found in the gland.

**Table 3:** Metastatic status of contralateral axillary lymph nodes.

	Level 1 lymph nodes	Level 2 lymph nodes	Level 3 lymph nodes
1	9/12	2/2	—
2	6/13	0/4	—
3	5/14	0/4	—
4	5/10	1/2	0/6
5	4/8	1/2	—
6	3/5	7/7	3/5
7	1/16	0/2	0/2
8	1/14	—	0/1
9	1/12	0/3	—
10	1/12	—	—
11	1/10	—	—
12	1/11	0/2	0/1

**Figure 2:**
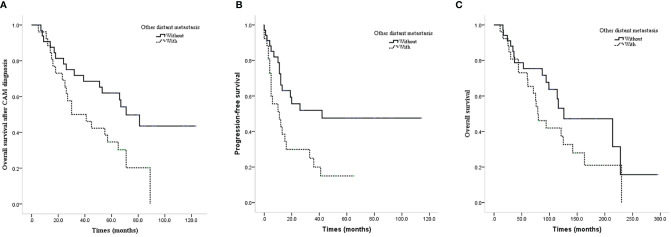
Survival curves of CAM patients with or without other distant metastases. **(A)** CAM-OS of CAM patients with or without other distant metastases. **(B)** PFS of CAM patients with or without other distant metastases. **(C)** OS of CAM patients with or without other distant metastases.

The prognosis of isolated CAM patients was better than that of patients with other distant metastases in terms of CAM-OS and PFS with significant differences (median CAM-OS 71.0 vs. 30.0 months, P=0.022; median PFS 42.0 vs. 11.0 months, P=0.009) and OS without significant differences (median OS 126.0 vs. 79.0 months, P=0.111, **Figures 2A**–**C**).

For the isolated CAM patients, 22 patients developed tumor progression after CAM treatment with a mean PFS of 34.4 months, and 18 patients survived during the follow-up. The five-year survival rate of isolated CAM patients was 67.4%, and the five-year disease-free survival (DFS) rate was 52.9%.

**Figure 3:**
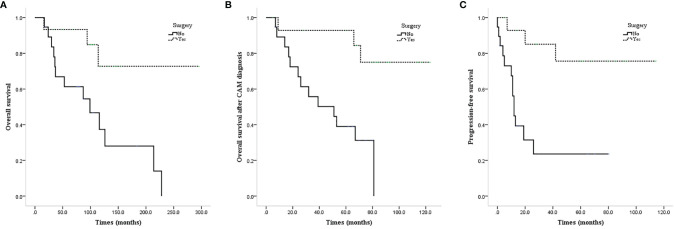
Survival curves of CAM patients based on undergoing axillary surgery or not. **(A)** OS of CAM patients based on undergoing axillary surgery or not. **(B)** CAM-OS of CAM patients based on undergoing axillary surgery or not. **(C)** PFS of CAM patients based on undergoing axillary surgery or not.

**Figure 4:**
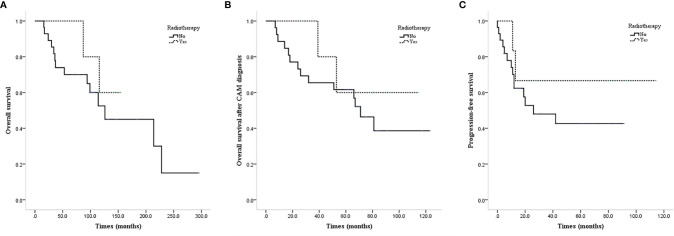
Survival curves of CAM patients based on undergoing axillary radiotherapy or not. **(A)** OS of CAM patients based on undergoing axillary radiotherapy or not. **(B)** CAM-OS of CAM patients based on undergoing axillary radiotherapy or not. **(C)** PFS of CAM patients based on undergoing axillary radiotherapy or not.

ALND or low-middle level ALND significantly improved the OS (mean OS 237.4 vs. 111.0 months, P=0.011; **Figure 3A**), CAM-OS (mean CAM-OS 105.2 vs. 46.6 months, P = 0.002; **Figure 3B**), and PFS (mean PFS 92.3 vs. 26.9 months, P = 0.001; **Figure 3C**) of isolated CAM patients. Axillary radiotherapy improved PFS, CAM-OS, and OS but without a significant difference (mean PFS 80.0 vs. 46.6 months, P = 0.345; mean CAM-OS 86.8 vs. 72.1 months, P = 0.338; mean OS 147.6 vs. 133.0 months, P = 0.426; **Figures 4A**–**C**).

### Comparison of Prognosis Between N3M0 and CAM Patients

A total of 538 patients who were initially diagnosed with N3M0 were screened, and the molecular type was clear in 478 patients. Case–control matching was performed between the 17 CAM patients with definite molecular types of the contralateral axillary lymph nodes and N3M0 patients with definite molecular types by molecular type, year of diagnosis ±2, and age of diagnosis ±2. Finally, a total of 16 pairs of patients were successfully matched 1:1.

**Figure 5:**
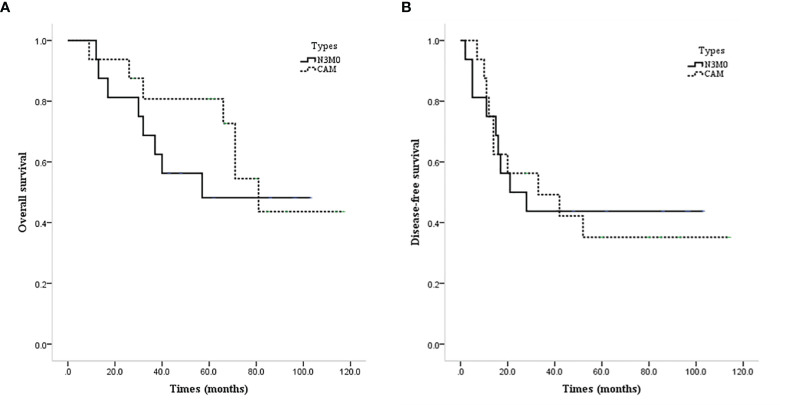
Survival curves of CAM and N3M0 patients. **(A)** OS of CAM and N3M0 patients. **(B)** DFS of CAM and N3M0 patients.

The prognosis of CAM patients after diagnosis of CAM was similar to that of N3M0 patients after initial diagnosis in terms of OS (mean OS 82.4 vs. 65.6 months, P=0.537, **Figure 5A**) and DFS (mean DFS 54.5 vs. 52.6 months, P=0.888, **Figure 5B**).

## Discussion

Although ipsilateral axillary lymph node metastasis is relatively common in breast cancer, CAM is rare. CAM can be classified into synchronous and metachronous CAM. The former, which is much rarer, exists when the primary tumor is diagnosed, and the latter appears after the treatment of the primary tumor (7, 9).

There are 3 possible sources for CAM as follows: 1) contralateral metastasis from the primary breast cancer; 2) metastasis from an occult primary in the ipsilateral breast; and 3) cancerization and metastasis from an extramammary site, such as adenocarcinoma of the uterus, gastrointestinal tract, ovary, thyroid, kidney, lymphoma, melanoma, squamous cell carcinoma of lung, squamous cell carcinoma of skin, or neurogenic tumor (10).

Ultrasound and MRI are performed on the ipsilateral breast of the CAM to determine whether there is a second primary tumor. The accuracy of MRI is higher than that of ultrasound. FDG PET/CT and lymphoscintigraphy are also used to detect the contralateral axillary lymph node metastasis of a second primary tumor (1). New breast primary tumors are in 33–75% of cases after resection of the ipsilateral breast of the CAM and careful pathological sectioning (10).

The actual incidence rate of CAM is difficult to assess. On the one hand, it is difficult to assess whether there is occult breast cancer due to a lack of magnetic resonance imaging, leading to the overestimated morbidity of CAM. On the other hand, some patients are unwilling to be reviewed or lost to follow-up, resulting in underestimated morbidity.

Ipsilateral supraclavicular lymph node metastasis was regarded as distant metastasis before the sixth edition of the AJCC cancer staging manual. However, Brito et al. (11) have shown that the DFS and OS of patients with ipsilateral supraclavicular lymph node metastasis are similar to those of patients with stage IIIB disease and significantly better than those of patients with stage IV disease. Therefore, ipsilateral supraclavicular lymph node metastasis is divided into locoregional metastasis. In the present study, we found that the DFS and OS of CAM patients were similar to those of N3M0 patients and significantly better than those of patients with other distant metastases.

The occurrence of CAM is closely related to the degree of malignancy on primary tumor histopathology and changes in the lymphatic drainage pathway (4). The changes in the physiological lymphatic drainage pathway can be caused by tumor invasion of the skin, blockage of lymphatic vessels by tumor thrombi, injury caused by radiotherapy, or surgical treatment by autopsy. Haagensen et al. (12) postulated that there may be deep lymphatic drainage through the deep fascia of the chest wall to the contralateral axillary. Using lymphography, the change in the lymphatic drainage pathway to the contralateral lymph nodes (such as axillary, internal mammary, or supraclavicular lymph nodes) can be found after breast or axillary surgery. Among 330 patients, Tokmak et al. (13) showed that 2 cases (0.6%) had lymphatic imaging of the contralateral axilla. Lizarraga et al. (14) demonstrated that 7.5% (8/107) of the patients who did not undergo surgery had contralateral lymphatic imaging. Lymphatic drainage outside the ipsilateral axillary fossa existed in 20-57% of primary breast cancer patients and in 0-2% of all patients at the initial diagnosis (8). However, the proportion was 18-70% after a previous operation or radiotherapy of the breast or axilla, and 14.7% of patients had contralateral axillary lymph drainage (8).

The occurrence of distant metastasis arises from circular tumor cells in the body. The change in lymphatic drainage may suggest that CAM is a local rather than a systemic manifestation (1, 15). The change in lymphatic drainage is more important than the invasiveness of tumors for CAM (9). According to this theory, CAM could be treated actively rather than conservatively.

Wang et al. (2) observed that CAM is associated with aggressive tumors and has poor prognosis, and they suggested that CAM is more likely to be distant metastases through lymphatic routes than local metastases of second primary tumors. CAM is well controlled by comprehensive treatment, including chemotherapy and radiotherapy, while the effect of axillary lymph node resection is insufficient. Mastectomy is not recommended for CAM. However, Gingerich et al. (9) suggested that CAM is secondary to lymphatic rather than hematogenous spread, according to the altered lymphatic drainage and aberrant pathways caused by surgery or radiotherapy. Based on this theory, the treatment of CAM can be aggressive and curative rather than palliative. We found that level 1 CAM was prior to that of level 2 and level 3 CAM, indicating that CAM may occur through chest wall lymphatic drainage rather than deep lymphatic drainage.

Chemotherapy and indicated endocrine therapy are indispensable if distant metastasis is considered or potential micrometastasis through skin lymphatic drainage outside the area of surgery and radiotherapy is needed for treatment (16). Morcos et al. (3) showed that the median DFS of 7 patients who only received endocrine therapy reached 24 months, including the longest time of 45 months of 1 case. Wang et al. (2) found that patients may obtain more survival benefits from chemotherapy or endocrine therapy than ALND or mastectomy. Neoadjuvant chemotherapy may test the sensitivity of treatment and improve the resectability of surgery (16). The PFS of CAM patients was significantly improved by radiotherapy (10 vs. 22 months) because radiotherapy can treat occult breast cancer on the same side and eradicate potential minute lesions in dermal lymphatics that may spread from the contralateral primary tumor (2). Because anti-HER2 treatment greatly improved the prognosis of breast cancer patients with CAM (3), anti-HER2 treatment should be considered in the treatment strategy of HER2-enriched cases (17, 18).

Surgery followed by radiotherapy is a reasonable and feasible scheme for patients without distant metastasis (16). Contralateral mastectomy is not recommended for the low incidence of contralateral occult breast cancer, except for some special cases, such as genetic breast cancer and CAM with different pathological and immunohistochemical features from the primary tumor (2, 3). None of the 9 patients who underwent ALND had axillary lymph node recurrence during the follow-up of 48 months (3, 10), and Huston et al. (10) showed that the DFS times of 2 patients after ALND were 29 months and 32 months. However, Wang et al. (2) considered that ALND was not effective because there was no statistical significance. The condition of the primary tumor and the timing of CAM should be considered in the formulation of therapeutic strategies (19).

Due to the insufficient number of cases and unclear immunohistochemical results, the effects of chemotherapy and endocrine and targeted therapy were not statistically analyzed. However, we still suggest that patients should receive more aggressive treatment to improve prognosis. We recommend axillary surgery for isolated CAM patients due to the improved local control and prolonged survival. In general, we do not recommend contralateral mastectomy because there was no lesion found in the resected breasts of all five patients. Although there was no significant difference, we suggest that radiotherapy should be performed to improve prognosis.

The survival time of patients with CAM varied. Most of the patients with synchronous CAM were at an advanced stage and had a worse prognosis than patients with early breast cancer, but patients with metachronous CAM had a longer survival time if there was no distant metastasis diagnosed. Chkheidze et al. (7) found that the mean CAM-OS was 27 months (range 7-40 months), the median OS after primary diagnosis was 32 months (range 13-124 months), and the median interval from CAM to distant metastasis was 18.5 months (range 5-33 months). The mean 5-year OS rate was 23% in patients with bone metastases and only 13% in patients with visceral metastasis (20). At present, Magnoni et al. (19) and Nash et al. (21) confirmed that the prognosis of CAM is not similar to that of metastatic breast cancer, but Guru et al. (22) suggested that CAM is comparable to stage IV disease in its natural history.

Moossdorff et al. (8) showed that 22 of 48 patients were followed up for a mean time of 50.3 months with an OS of 82.6% and a DFS of 65.2%. The mean follow-up time was 69.2 months for patients with isolated CAM, and the OS and DFS were 76.9% and 46.1%, respectively. Magnoni et al. (19) reported that the estimated OS was 72% at 5 years (95% CI 54–83), and the estimated DFS was 61% at 5 years (95% CI 44–74). The prognosis of patients with CAM was better than that of patients with distant metastasis, and it was comparable to that of patients with regional recurrence (5-year DFS, 56-84%). Postlewait et al. (23) showed that inflammatory breast cancer patients with isolated CAM had statistically similar survival to those with stage III disease. Therefore, it seems unjustified to classify CAM as distant metastasis.

In our analysis, the prognosis of CAM was much better than that of distant metastasis and similar to that of N3M0. This is the first study to directly compare the prognosis of N3M0 and CAM patients. At the same time, the five-year survival and five-year DFS rates of isolated CAM were similar to those of local recurrence, indicating that CAM should be considered local recurrence rather than distant metastasis.

Our study was a retrospective analysis with few patients included, and there were some restrictions for the analysis results. The subgroup analysis was limited due to insufficient cases, and the analysis results may be biased due to individual cases. Owing to the long span of cases and the change in treatment concept, the patients included later were treated more actively, resulting in the deviation of subgroup analysis.

## Conclusion

A systemic examination should be completed to assess the status of lymph nodes for patients with recurrent breast cancer, especially for the contralateral axillary lymph nodes. Breast MRI should be completed to exclude occult breast cancer for CAM patients. Because the prognosis of CAM patients is similar to that of N3M0 patients and significantly better than that of patients with distant organ involvement, CAM should be treated as local recurrence with aggressive and curative rather than palliative strategies. The prognosis of CAM patients could be improved by ALND or low-middle level ALND and radiotherapy. Most isolated CAM will develop into distant metastasis combined with other sites, and active comprehensive treatment can control disease progression more effectively.

## Data Availability Statement

The raw data supporting the conclusions of this article will be made available by the authors, without undue reservation.

## Ethics Statement

The studies involving human participants were reviewed and approved by Ethics Committee of the Affiliated Cancer Hospital of Shandong First Medical University. The patients/participants provided their written informed consent to participate in this study. Written informed consent was obtained from the individual(s) for the publication of any potentially identifiable images or data included in this article.

## Author Contributions

LZ: manuscript writing, data collection, and statistical analysis. XW: manuscript writing, data collection, and statistical analysis. CL: revision and manuscript writing. QY: revision and manuscript writing. ZL: data collection and manuscript reviewing. ZY: guarantor, revision, manuscript writing, and supervision. The authors read and approved the final manuscript.

## Conflict of Interest

The authors declare that the research was conducted in the absence of any commercial or financial relationships that could be construed as a potential conflict of interest.

## Publisher’s Note

All claims expressed in this article are solely those of the authors and do not necessarily represent those of their affiliated organizations, or those of the publisher, the editors and the reviewers. Any product that may be evaluated in this article, or claim that may be made by its manufacturer, is not guaranteed or endorsed by the publisher.
